# A Case-Control Study of the Association Between the SPP1 Gene SNPs and the Susceptibility to Breast Cancer in Guangxi, China

**DOI:** 10.3389/fonc.2019.01415

**Published:** 2019-12-20

**Authors:** Lina Liang, Guanming Lu, Guogang Pan, Yibin Deng, Jiadong Liang, Limei Liang, Jia Liu, Yujin Tang, Guijiang Wei

**Affiliations:** ^1^Department of Medical Laboratory, Affiliated Hospital of Youjiang Medical University for Nationalities, Baise, China; ^2^Department of Breast and Thyroid Surgery, Affiliated Hospital of Youjiang Medical University for Nationalities, Baise, China; ^3^Department of Spinal Surgery, Affiliated Hospital of Youjiang Medical University for Nationalities, Baise, China

**Keywords:** breast cancer, secreted phosphoprotein-1, single nucleotide polymorphism, allele, risk

## Abstract

Secreted phosphoprotein-1 (SPP1) has been reported to be involved in the pathogenesis of breast cancer (BRC), but the influence of SPP1 single nucleotide polymorphisms on the BRC susceptibility has been rarely reported. In this study, we explored the association between rs11730582, rs2853750, and rs35893069 in the SPP1 gene and the BRC susceptibility. We used Snapshot assay to detect SPP1 single nucleotide polymorphisms in 471 BRC patients and 471 controls. The plasma SPP1 level was measured by ELISA. We found that the CC genotype and C allele of rs11730582 were associated with a significantly decreased BRC risk compared with the TT genotype and T allele, respectively [CC vs. TT: odds ratio (OR) = 0.59, 95% CI = 0.37–0.94, *P* = 0.026; C vs. T: OR = 0.79, 95% CI = 0.65–0.96, *P* = 0.022]. In addition, BRC patients and controls with the rs11730582 CC genotype had a lower plasma SPP1 level than did BRC patients and controls with TT genotype (*P* = 0.007 and *P* = 0.011, respectively). Moreover, the proportions of rs11730582 CC genotype and C allele were decreased in BRC patients with clinical stages I–III compared with those with clinical stage IV (*P* = 0.012 and *P* = 0.003, respectively). Besides, the C-G-T haplotype was associated with a significantly decreased BRC risk compared with the T-A-T haplotype (OR = 0.69, 95% CI = 0.52–0.93, *P* = 0.015). However, there was no significant association between rs2853750 or rs35893069 and the BRC risk. In summary, our study found the association between rs11730582 and the risk of BRC and suggested that rs11730582 may promote the occurrence and development of BRC by regulating SPP1 expression.

## Introduction

Breast cancer (BRC) is the most common cancer type and a leading cause of mortality among females in the world (1). According to the statistics from the American Cancer Society, the BRC incidence in the United States was 46.3 per 100 thousand females and the mortality was 13.0 per 100 thousand females (2). In India, BRC ranked first among different types of cancer, with the incidence of 25.8 per 100 thousand females and the mortality of 12.7 per 100 thousand females (3). China has the largest number of BRC patients in the world. About 278 thousand females were diagnosed as having BRC in 2014; the incidence was 30.6 per 100 thousand females and the mortality was 6.5 per 100 thousand females (4, 5). Because of the high incidence and mortality, BRC has brought numerous families in the world a great economic and health burden (6, 7). The etiology of BRC is involved in multiple factors including smoking, diet, estrogen exposure, irregularity of menstruation periods, family history of BRC, and so on (8, 9). However, these risk factors just clarify some parts of the BRC pathogenesis, and the underlying etiology of BRC still needs to be further elucidated.

Secreted phosphoprotein-1 (SPP1), also known as osteopontin, is an extracellular matrix protein that is produced in multiple human tissues and involved in the control of biomineralization and calcification (10, 11). Some studies suggested that SPP1 played an important role in the progression and metastasis of several types of cancers. In a study about ovarian cancer, SPP1 was found to be overexpressed in ovarian cancer tissues and promote ovarian cancer progression (12). The overexpression of SPP1 was also found in colorectal cancer tissues and related to poor prognosis (13). Recently, Pio et al. reported that SPP1 was up-regulated in the BRC cells and promoted their migration and stem-like behavior, potentially through activation of the WNK-1 and PRAS40-related pathways (14). Similarly, SPP1 was also found to be increased in normal human breast tissue at a high risk of developing BRC (15) and promote inflammation and tumor growth by reprograming normal mammary fibroblasts (16). The studies above suggested that SPP1 was a potential biomarker or a novel therapeutic target for BRC.

Previous works had shown that single nucleotide polymorphisms (SNPs) in the SPP1 gene were associated with the risk or metastasis of some human cancers, such as oral carcinogenesis (17), hepatocellular carcinoma (18), and BRC (19). Particularly, the association between SNPs in the SPP1 gene and the susceptibility to BRC had been evaluated among the population in Egypt, but the sample size was small (60 cases and 60 controls) (20). A further study with a larger sample size should be conducted immediately to reveal the association between SNPs in the SPP1 gene and the susceptibility to BRC. Therefore, we conducted this study among the population in Guangxi, China. In our study, we genotyped SNPs in the SPP1 gene, measured its expression level, and assessed the impact of SNPs in the SPP1 gene on the expression level of SPP1 and other BRC-related characteristics.

## Materials and Methods

### Study Population

Four hundred seventy-one histologically confirmed BRC patients were recruited in the Department of Breast and Thyroid Surgery, Affiliated Hospital of Youjiang Medical University for Nationalities from February 2017 to November 2018. Clinical information was collected from medical records including age at diagnosis, age at menarche, estrogen receptor status, progesterone receptor status, and clinical stages. Patients who had a family history of cancer, recurring BRC, or BRC combined with other types of cancers were excluded. Four hundred seventy-one controls were healthy volunteers visiting the same hospital from December 2017 to December 2018. We excluded those controls who had a family history of cancer, breast lesions, or breast mastitis. All the subjects came from Guangxi, China. About 2–3 ml anticoagulant whole blood sample was taken from each participant. The study was approved by the Ethics Committee of Youjiang Medical University for Nationalities, conformed to the Declaration of Helsinki, and received written informed consent from all subjects before the study.

### SNP Selection and Genotyping

We used Promoter 2.0 Prediction Server (http://www.cbs.dtu.dk/services/Promoter/) and The Eukaryotic Promoter Database (http://www.epd.isb-sib.ch/) to predict promoter. Subsequently, SNPs with minor allele frequency (MAF) >30% among the Southern Han Chinese population were selected by searching in the promoter and open reading frame of the SPP1 gene in the National Center for Biotechnology Information SNP database (http://www.ncbi.nlm.nih.gov/SNP) and 1000 Genomes Project (http://browser.1000genomes.org). Finally, we evaluated the association between three SNPs (rs11730582, rs2853750, and rs35893069) in the SPP1 gene and the susceptibility to BRC. The basic information of the three SNPs is listed in Supplementary Table 1.

DNA extraction kit (Tiangen, China) was used to extract DNA from blood samples and then stored the DNA at −80°C. Primers were designed by Primer 5 (PREMIER Biosoft) and synthesized by Sangon Company, and are listed in Supplementary Table 2. After amplifying the SPP1 gene by polymerase chain reaction, the three SNPs were genotyped by Snapshot assay.

### Plasma SPP1 Level Determination

Plasma was separated from whole blood samples of patients and controls at room temperature and stored at −70°C until analysis. Plasma SPP1 level was determined according to the instruction of ELISA kit (BioVision, Catalog: K7335). The developed color reaction was measured at 450-nm wavelength on the ELISA reader (Bio-Rad, USA), and detection range was from 0.2 to 70 ng/ml.

### Statistics

Statistics were mainly proceeded by Statistical Package for Social Science 21. The percentages were calculated for clinical features and different genotypes and alleles, and the means and SDs were calculated for age at diagnosis and age at menarche. The data normality for continuous variables was assessed by Shapiro-Wilk tests, then the continuous variables were analyzed by the independent-sample *t*-test. We performed the Hardy-Weinberg equation for the three SNPs in our study. Two-sided chi-square test was used to assess the conformity to the Hardy-Weinberg rule of the three SNPs genotypes. Categorical variables comparisons were also proceeded by two-sided chi-square test. The associations of the three SNPs with BRC susceptibility were assessed in genotypic, allelic, recessive (rs11730582: CC + TC vs. TT; rs2853750: GG + AG vs. AA; rs35893069: TT + AT vs. AA), and dominant model (rs11730582: CC vs. TC + TT; rs2853750: GG vs. AG + AA; rs35893069: TT vs. AT + AA). Logistic regression was used for exploring the association between SPP1 SNPs and the susceptibility to BRC, and *P* values, odds ratio (OR), and 95% CI were adjusted by age at diagnosis and age at menarche. Genetical distribution of rs11730582 among 11 different populations were collected on the website below (http://www.internationalgenome.org). Haplotype analysis was proceeded by SHEsis Main (http://analysis.bio-x.cn/). *P* < 0.05 was regarded as statistically significant. The GAS Power Calculator was used for power calculation, which was proceeded by rs11730582 with the minimum MAF among the three SNPs. Under the conditions of sample size (cases = 471, controls = 471), disease prevalence = 0.00041 (4), significance level = 0.05, MAF = 0.31, and OR = 1.5), we had 98% statistical power in this study.

## Results

### Clinical Features of Cases and Controls

Clinical features of cases and controls are shown in Table 1. As for the distribution of age at diagnosis and age at menarche, there was no significant difference between BRC patients and the controls (*P* = 0.630 and *P* = 0.596, respectively). The proportions of cases with positive estrogen receptor, positive progesterone receptor, and III–IV clinical stage were 61.9, 55.0, and 37.1%, respectively.

**Table 1:** Clinical features of cases and controls.

**Variables**	**Controls (*n* = 471)**	**Cases (*n* = 471)**
Age at diagnosis, years (mean ± SD)	47.6 ± 9.2	47.9 ± 9.5
Age at menarche, years (mean ± SD)	14.1 ± 1.0	14.0 ± 1.3
Estrogen receptor (%)		
Positive		292 (61.9)
Negative		179 (38.1)
Progesterone receptor (%)		
Positive		259 (55.0)
Negative		212 (45.0)
Clinical stages (%)		
I–III		397 (84.3)
IV		74 (15.7)

### Association Between the Three SPP1 SNPs and the Risk of BRC

As shown in Supplementary Table 1, the genotypes of rs11730582, rs2853750, and rs35893069 conformed to the Hardy-Weinberg rule (*P* = 0.685, *P* = 0.973, and *P* = 0.966, respectively). The association between the three SPP1 SNPs (rs11730582, rs2853750, and rs35893069) and the risk of BRC is shown in Table 2. We found that the rs11730582 CC genotype was associated with a significantly decreased BRC risk compared with the TT genotype (OR = 0.59, 95% CI = 0.37–0.94, *P* = 0.026). Similarly, we also observed a significantly decreased risk in allele analysis (OR = 0.79, 95% CI = 0.65–0.96, *P* = 0.022). However, no significant association of the rs2853750 and rs35893069 with the risk of BRC was found (*P* > 0.05).

**Table 2:** Association between SPP1 SNPs and the risk of BRC.

**SNPs**	**Controls**	**Cases**	**OR**	***P*-value^*†*^**
	**(*n* = 471)**	**(*n* = 471)**	**(95% CI)^*†*^**	
rs11730582				
TT	213 (45.2)	241 (51.2)	1.00 (reference)	
TC	204 (43.3)	194 (41.2)	0.84 (0.64–1.09)	0.208
CC	54 (11.5)	36 (7.6)	0.59 (0.37–0.94)	**0.026**
CC + TC vs. TT			0.79 (0.61–1.02)	0.070
CC vs. TC + TT			0.64 (0.41–1.01)	0.052
T	630 (66.9)	676 (71.8)	1.00 (reference)	
C	312 (33.1)	266 (28.2)	0.79 (0.65–0.96)	**0.022**
rs2853750				
AA	143 (30.4)	148 (31.4)	1.00 (reference)	
AG	234 (49.7)	232 (49.3)	0.95 (0.71–1.28)	0.782
GG	94 (19.9)	91 (19.3)	0.93 (0.64–1.34)	0.729
GG + AG vs. AA			0.95 (0.72–1.25)	0.733
GG vs. AG + AA			0.96 (0.70–1.33)	0.817
A	520 (55.2)	528 (56.1)	1.00 (reference)	
G	422 (44.8)	414 (43.9)	0.96 (0.80–1.16)	0.723
rs35893069				
AA	132 (28.0)	136 (28.9)	1.00 (reference)	
AT	242 (51.4)	234 (49.7)	0.94 (0.69–1.26)	0.660
TT	97 (20.6)	101 (21.4)	1.01 (0.70–1.44)	0.991
TT + AT vs. AA			0.96 (0.72–1.28)	0.761
TT vs. AT + AA			1.06 (0.77–1.44)	0.729
A	506 (53.7)	506 (53.7)	1.00 (reference)	
T	436 (46.3)	436 (46.3)	1.01 (0.84–1.20)	0.970

*OR, odds ratio; SPP1, secreted phosphoprotein-1; SNP, secreted phosphoprotein-1; BRC, breast cancer*.

† *Adjusted by age at diagnosis and age at menarche*.

*P < 0.05 was indicated in bold*.

### Association Between rs11730582 and Plasma SPP1 Level

**Figure 1:**
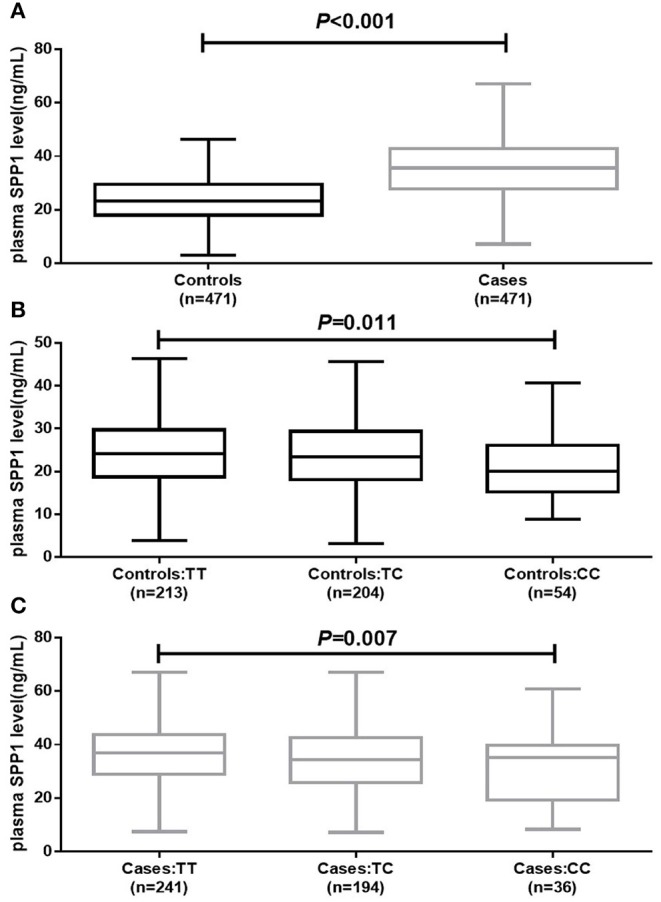
Plasma secreted phosphoprotein-1 (SPP1) level in controls and cases. **(A)** Decreased plasma SPP1 level was found in breast cancer (BRC) patients compared to controls (*P* < 0.001). **(B)** Decreased plasma SPP1 level was found in controls carrying the rs11730582 CC genotype compared with those carrying the rs11730582 TT (*P* = 0.011). **(C)** Decreased plasma SPP1 level was found in BRC patients carrying the rs11730582 CC genotype compared with those carrying the rs11730582 TT (*P* = 0.007). The upper and lower boundaries of the boxes represented the 25th and 75th percentiles of plasma SPP1 level value, and the horizontal line within the box represents the median value. All *P*-values were adjusted by age at diagnosis and age at menarche.

Association between rs11730582 and plasma SPP1 level is shown in Figure 1. Plasma SPP1 level was significantly up-regulated in BRC patients compared with controls (*P* < 0.001). Moreover, we found that BRC patients with the rs11730582 CC genotype had a lower plasma SPP1 level than did those with the TT genotype (*P* = 0.007). Similarly, controls with the rs11730582 CC genotype also had a lower plasma SPP1 level than did those with the TT genotype (*P* = 0.011). Nevertheless, there was no significant difference between individuals with the rs11730582 CC genotype and those with the TC genotype on plasma SPP1 level.

### Association Between rs11730582 and BRC Clinical Features

Association between rs11730582 and BRC clinical features is shown in Table 3, and the proportion of rs11730582 CC genotype was decreased in BRC patients with clinical stages I–III compared with those with clinical stage IV (*P* = 0.012). Besides, the rs11730582 A allele was also decreased in BRC patients with clinical stages I–III compared with those with clinical stage IV (*P* = 0.003). However, there was no significant difference between rs11730582 and estrogen receptor status or progesterone receptor status of BRC patients.

**Table 3:** The associations between rs11730582 and clinical features of BRC.

**Variables**	***n***	**Genotypes (%)**			***P*-value^*†*^**	**Alleles (%)**		***P*-value^*†*^**
		**TT**	**TC**	**CC**		**T**	**C**	
Estrogen receptor								
Positive	292	148 (50.6)	121 (41.5)	23 (7.9)		417 (71.4)	167 (28.6)	
Negative	179	93 (51.9)	73 (40.8)	13 (7.3)	0.943	259 (72.3)	99 (27.7)	0.739
Progesterone receptor								
Positive	259	141 (54.4)	101 (38.9)	17 (6.7)		383 (73.9)	135 (26.1)	
Negative	212	100 (47.2)	93 (43.9)	19 (8.9)	0.267	293 (69.1)	131 (30.9)	0.108
Clinical stages								
I–III	397	192 (48.4)	171 (43.1)	34 (8.5)		555 (69.9)	239 (30.1)	
IV	74	49 (66.2)	23 (30.1)	2 (2.7)	**0.016**	121 (81.8)	27 (12.2)	**0.003**

† *Adjusted by age at diagnosis and age at menarche*.

*P < 0.05 was indicated in bold*.

### Haplotype Analysis

Haplotype analysis of rs11730582-rs2853750-rs35893069 with the BRC risk is shown in Table 4; six haplotypes were listed, rs11730582 was in a strong linkage disequilibrium with rs2853750 (D′ = 0.981) and rs35893069 (D′ = 0.810), and rs2853750 was also in a strong linkage disequilibrium with the rs35893069 (D′ = 0.849). Moreover, T-A-T and T-A-A were two major haplotypes in both controls (30.5 and 24.6%, respectively) and BRC patients (31.7 and 24.3%, respectively). In addition, we observed that the C-G-T haplotype was associated with a significantly decreased BRC risk compared with the T-A-T haplotype (OR = 0.69, 95% CI = 0.52–0.93, *P* = 0.015).

**Table 4:** Haplotype analysis of rs11730582-rs2853750-rs35893069 with the BRC risk.

**Haplotype**	**Controls (2*n* = 942)**	**Cases (2*n* = 942)**	**OR (95% CI)**	***P*-value**
T-A-T	287 (30.5)	299 (31.7)	1.00 (reference)	
T-A-A	232 (24.6)	229 (24.3)	0.94 (0.74–1.21)	0.665
C-G-A	164 (17.4)	159 (16.9)	0.93 (0.71–1.22)	0.604
C-G-T	148 (15.7)	107 (11.4)	0.69 (0.52–0.93)	**0.015**
T-G-A	110 (11.7)	118 (12.5)	1.03 (0.76–1.40)	0.851
T-G-T	1 (0.1)	30 (3.2)	–	–

*P < 0.05 was indicated in bold*.

### Genetical Distribution of rs11730582 Among 11 Different Populations

Genetical distribution of rs11730582 among 11 different populations is shown in Table 5. The genetical distribution of rs11730582 in this study was significantly different from those among 1KGP-ACB, 1KGP-CLM, 1KGP-ASW, 1KGP-PEL, 1KGP-TSI, 1KGP-PJL, and 1KGP-CEU populations (*P* < 0.05). In contrast, there was no significant difference after comparing with 1KGP-CHB, 1KGP-CHS, and 1KGP-JPT populations (*P* > 0.05).

**Table 5:** Genotypic and allelic distributions of rs11730582 among different populations.

**Populations**	***n***	**Genotypes (%)**			***P*-value**	**Alleles (%)**		***P*-value**
		**TT**	**TC**	**CC**		**T**	**C**	
Present data	471	213 (45.2)	204 (43.3)	54 (11.5)		630 (66.9)	312 (33.1)	
1KGP-CHS	105	50 (47.6)	45 (42.9)	10 (9.5)	0.817	145 (69.0)	65 (31.0)	0.545
1KGP-CHB	103	46 (44.7)	45 (43.7)	12 (11.7)	0.994	137 (66.5)	69 (33.5)	0.918
1KGP-JPT	104	36 (34.6)	53 (51.0)	15 (14.4)	0.139	125 (60.1)	83 (39.9)	0.062
1KGP-ACB	96	71 (74.0)	23 (24.0)	2 (2.0)	**<0.001**	165 (85.9)	27 (14.1)	**<0.001**
1KGP-ASW	61	48 (78.7)	12 (19.7)	1 (1.6)	**<0.001**	108 (88.5)	14 (11.5)	**<0.001**
1KGP-CLM	94	30 (31.9)	39 (41.5)	25 (26.6)	**<0.001**	99 (52.7)	89 (47.3)	**<0.001**
1KGP-PEL	85	25 (29.4)	45 (52.9)	15 (17.6)	**0.019**	95 (55.9)	75 (44.1)	**0.006**
1KGP-TSI	107	35 (32.7)	51 (47.7)	21 (19.6)	**0.018**	121 (56.5)	93 (43.5)	**0.004**
1KGP-CEU	99	24 (24.2)	54 (54.5)	21 (21.3)	**<0.001**	102 (51.5)	96 (48.5)	**<0.001**
1KGP-PJL	96	29 (30.2)	48 (50.0)	19 (19.8)	**0.010**	106 (55.2)	86 (44.8)	**0.002**

*1KGP, 1000 genomes project; CHS, Southern Han Chinese; CHB, Han Chinese in Beijing; JPT, Japanese in Tokyo; ACB, African Caribbean in Barbados; ASW, African Ancestry in Southwest USA; CLM, Colombian in Medellin; PEL, Peruvian in Lima; TSI, Toscani in Italy; CEU, Utah residents with Northern and Western European ancestry; PJL, Punjabi in Lahore. P < 0.05 was indicated in bold*.

## Discussion

There are millions of females suffering from BRC in the world; it has brought numerous families an unaffordable economic burden (6, 7). SPP1, a tumor-related extracellular matrix protein, has been reported to be involved in the pathogenesis of BRC (10, 11). Pio et al. reported that SPP1 was up-regulated in BRC cells and promoted their migration and stem-like behavior (14). Similar result was found in a study conducted by Lindahl et al. (15). In our study, the upregulation of plasma SPP1 level in BRC patients was also observed and consistent with the results of previous studies.

SNPs have been broadly reported to cause the abnormal functions of various genes. Until now, a few studies had focused on the association of SPP1 SNPs with the susceptibility to some human cancers. Chiu et al. reported that a less prevalence of the rs11730582 CC genotype was found in oral squamous cell carcinoma patients from Taiwan (17). Likewise, Dong et al. reported that hepatocellular carcinoma patients with the rs11730582 CC genotype had a longer overall survival and time to recurrence compared to those with the rs11730582 TT/TC genotype (18). Notably, Ramchandani and Weber corroborated that the rs11730582 C allele was associated with lower aggressiveness of BRC (19). In addition, in a study performed by Zakhary et al., the rs11730582 CC genotype was found to be significantly lower in BRC patients compared to controls among the Egyptian population, but the sample size was too small (60 cases and 60 controls) (20). In our study, we found that the rs11730582 CC genotype and C allele were associated with a significantly decreased BRC risk compared with the TT genotype and T allele, respectively. The results in our study were consistent with the studies above and indicated that rs11730582 may be involved in the BRC etiology. The association of rs2853750 and rs11730582 with the BRC risk was firstly reported. However, we found a negative association of rs2853750 or rs35893069 with the BRC risk in this study. There are some explanations for the negative result. Both rs2853750 and rs35893069 are the SNPs locating in the introns of SPP1 gene. Previous studies showed that an SNP in the intron could influence RNA splicing process during the maturation of mRNA (21, 22), but sometimes this effect was lost (23, 24). In addition, few important gene regulation-related elements locate in the introns of a gene.

In consideration of that rs11730582 located in the promoter of SPP1 gene, we further assessed SPP1 expression with the rs11730582 genotypes. In the present study, BRC patients with the rs11730582 CC genotype had a lower plasma SPP1 level than did those with the TT genotype. Similarly, controls with the rs11730582 CC genotype also had a lower plasma SPP1 level than did those with the TT genotype. Some previous studies supported our results. In a study performed by Ramchandani and Weber, the proportion of rs11730582 CC genotype was found to be decreased in BRC patients and controls with a high SPP1 level (19). Analogously, Zakhary et al. also found that the rs11730582 CC genotype was associated with a lower SPP1 level compared to the TT genotype in BRC patients (20). As we have known, SNPs in the promoter of a gene could alter the promoter transcription activity; we deduced that the rs11730582 genotypes might alter plasma SPP1 level by changing the transcription activity of SPP1 gene. The deduction was verified in a study conducted by Dong et al. (18). In their study, SPP1 level in hepatocellular carcinoma cell transfected with SPP1 promoter containing the rs11730582 CC genotype was significantly lower than that in hepatocellular carcinoma cell transected with the TT genotype.

We further investigated the association between rs11730582 and BRC clinical features. In the current study, the proportions of rs11730582 CC genotype and C allele were decreased in BRC patients with clinical stages I–III compared with those with clinical stage IV. Based on the published studies mentioned above, a significantly increased SPP1 level had been regarded as an important role to promote the development of BRC, and the individuals with rs11730582 CC genotype had a lower plasma SPP1 level than did those with TT genotype. Herein, we concluded that the rs11730582 CC genotype might downregulate the transcription activity of SPP1 gene and plasma SPP1 level, finally promoting the occurrence and development of BRC.

In the present study, we performed a haplotype analysis for rs11730582-rs2853750-rs35893069. We observed that the C-G-T haplotype was associated with a significantly decreased BRC risk compared with the major haplotype T-A-T. Similarly, in a study about sarcoidosis, Maver et al. found that a haplotype with three SPP1 SNPs including rs11730582 significantly decreased the sarcoidosis risk (25). In addition, Glas et al. reported that a haplotype with eight SPP1 SNPs including rs11730582 was strongly associated with the susceptibility to Crohn's disease (26). These studies above indicated that rs11730582-related SPP1 haplotypes might be closely associated with the BRC risk, and also prove the importance of rs11730582 in the BRC pathogenesis.

There are two limitations in our study. First, all cases and controls in our study came from Guangxi; the subjects from other regions of the world should be recruited. As shown in Table 5, the genetical distribution of rs11730582 among in the population of Guangxi was similar to those among 1KGP-CHB, 1KGP-CHS, and 1KGP-JPT populations, but significantly different from those among 1KGP-ACB, 1KGP-CLM, 1KGP-ASW, 1KGP-PEL, 1KGP-TSI, 1KGP-PJL, and 1KGP-CEU populations. The difference in genetical distribution among different populations caused the different susceptibility to BRC. Although our study firstly reported the susceptibility to BRC in the population of Guangxi and offered important evidence for BRC pathogenesis, it was still very important to investigate the susceptibility to BRC in other populations. In the future, further studies in other populations will be performed. Second, we investigated SNPs in the SPP1 gene in our study; the results provided a partial insight into the BRC pathogenesis. More BRC risk factors like epigenetics and so on should be investigated to understand the BRC pathogenesis thoroughly in future studies.

In conclusion, the present study found the association between SPP1 rs11730582 and the BRC risk among the population of Guangxi China, and suggested that rs11730582 may promote the occurrence and development of BRC by regulating SPP1 expression. The results may provide important evidence for the etiology of BRC and a potential BRC biomarker.

## Data Availability Statement

The datasets generated for this study can be found in the datasets in the current study are available from the corresponding author on reasonable request.

## Ethics Statement

The studies involving human participants were reviewed and approved by the Ethics Committee of Youjiang Medical University for Nationalities. The patients/participants provided their written informed consent to participate in this study. Written informed consent was obtained from the individual(s) for the publication of any potentially identifiable images or data included in this article.

## Author Contributions

LinL and GW designed the study and wrote the manuscript. LinL, GL, and GP performed experiments. YD, JLia, and LimL performed statistical analysis. JLiu and YT helped to revise the manuscript.

### Conflict of Interest

The authors declare that the research was conducted in the absence of any commercial or financial relationships that could be construed as a potential conflict of interest.
