# Supplementary material for: A Case-Control Study of the Association Between the SPP1 Gene SNPs and the Susceptibility to Breast Cancer in Guangxi, China
Source: Front Oncol. 2019 Dec 20;9:1415. doi: 10.3389/fonc.2019.01415 (PMC6933604; doi:10.3389/fonc.2019.01415)
Supplement: Supplementary file 1 [file Table_1.DOCX]

**Supplementary** **Table 1. The basic information of selected SNPs**

| **Rs number** | **Gene symbol** | **Allele position** | **Region** | **MAF** | ***P*_HWE_** |
| --- | --- | --- | --- | --- | --- |
| rs11730582 | SPP1 | Chromosome 4: 87975269 | Promoter | 0.310 | 0.685 |
| rs2853750 | SPP1 | Chromosome 4: 87976728 | Intron | 0.448 | 0.973 |
| rs35893069 | SPP1 | Chromosome 4: 87978282 | Intron | 0.457 | 0.966 |

MAF: minor allele frequency; HWE: Hardy–Weinberg equilibrium.

**Supplementary Table 2. Primers for genotyping SPP1 SNPs**

| **SNP loci** | **Sequences of PCR primers** | | **Sequences of extension primers** |
| --- | --- | --- | --- |
| rs11730582 | Upstream primer | 5’-ATACTCGAAATCACAAAGC-3’ | 5’-TTTTTTTTTTTTGCCTGCAAGGAGTTCAGA-3’ |
|  | Downstream primer | 5’-TTCAGCATCCAGGAAGAG-3’ |  |
| rs2853750 | Upstream primer | 5’-TGGATAATAGGTGGCAATA-3’ | 5’-TTTTTTTTTTTTTAACTTATACATCCATTCTCT-3’ |
|  | Downstream primer | 5’-TGTACTCACTGGTATGGC-3’ |  |
| rs35893069 | Upstream primer | 5’-TGAGTGTTAGGATTACTGGGTG-3’ | 5’-TTTTTTTTTTTTTTAATACAGGCTCAGAGAGAA-3’ |
|  | Downstream primer | 5’-GTGGATCACGAGGTCAGG-3’ |  |
